# Sensory attributes, chemical and microbiological properties of cigars aged with different media

**DOI:** 10.3389/fbioe.2023.1294667

**Published:** 2023-10-24

**Authors:** Wanrong Hu, Wen Cai, Yun Jia, Jingyuan Fan, Beibei Zhu, Qianying Zhang, Yue Wang, Yuanfa Liu, Dongliang Li

**Affiliations:** ^1^ Cigar Technology Innovation Center of China Tobacco, China Tobacco Sichuan Industrial Co., Ltd., Chengdu, China; ^2^ Cigar Fermentation Technology Key Laboratory of Tobacco Industry, China Tobacco Sichuan Industrial Co., Ltd., Chengdu, China; ^3^ Industry Efficient Utilization to Domestic Cigar Tobacco Key Laboratory of Sichuan Province, Shifang, China; ^4^ School of Food Science and Technology, Jiangnan University, Wuxi, China

**Keywords:** cigar, aging, medium, chemical composition, community succession, sensory quality

## Abstract

**Introduction:** Aging is an important process to improve the quality of cigar, but the effect of aging with media on cigar has not been reported.Therefore, this study aimed to prepare different aging media and clarify the influence of media on cigar quality.

**Methods:** Effective media were first screened by sensory evaluation, then the effects of aging media on the chemical composition and microbial community of cigar were investigated.

**Results:** The results showed that: 1) As aging media, coffee formula and cocoa formula could optimize the smoke and aroma characteristics of cigar, and 30 days was the appropriate period for cigar aging. 2) Aging with coffee or cocoa media could increase the content of amino acids, non-volatile organic acids, malic acid and aroma components. Particularly, the content of aroma components increased from 2.48 mg g^−1^ (W-30) to 3.21 mg g^−1^ (C-30) and 3.70 mg g^−1^ (K-30), respectively. 3) Aging with coffee can improve the diversity of bacteria and fungi on the cigar surface and change the succession rule of bacterial community. In contrast, aging with cocoa had no significant effect on microbial diversity of cigar.

**Discussion:** In this study, the influence of aging media on cigar quality was analyzed multidimensionally for the first time, which provided a reference for the development of new aging media and technologies to improve cigar quality.

## 1 Introduction

The main production process of cigars includes cultivation, air-drying, fermentation, rolling, and aging. Aging is an important process in cigar production, which occurs after rolling and can further reduce the irritation and improve the aftertaste of cigar (Feng et al., 2022). Unlike cigarettes, cigars show a relatively higher activity of microorganisms and enzymes after rolling, resulting in further biochemical reactions. As a result, the chemical components of cigars tend to be harmonized, and the sensory quality can be further improved after aging (Fan et al., 2023). In general, the aging of cigars should occur in a medium environment with the set temperature and relative humidity (20°C ± 2°C, 62% ± 2%). The most common aging medium is Spanish cedar (Fan et al., 2023). This is because Spanish cedar not only has excellent ability for moisture regulation, but also can prevent cigars from insects (Fan et al., 2016). Besides, Spanish cedar can endow cigar with unique cedar fragrance and ensure the stable quality of cigars during the long aging process. However, the single aging method could not meet the consumer demand for cigar flavor.

Recently, more attention has been paid on aging media of cigar, aiming to explore more novel aging media alternatives to Spanish cedar. Liu et al. (2022) had investigated the variation rule of volatile compounds in cigar aged with cypress or Chinese fir. The results showed that both wood treatments can significantly increase the variety of volatile components in cigar, and alkenes were the main volatile components, which significantly affect the quality and style characteristics of cigar. According to the traditional technology inheritance and experience accumulation, Great Wall Cigar Factory (China) has carried out research on different media, such as porcelain pot and oak barrel. In addition to solid media, liquid media such as clove-whiskey formula, cinnamon-maotai liquor formula, pepper-red wine formula, hazelnut-whiskey formula and green tea extract have also been used to enhance aroma and quality of cigar (Hu et al., 2023). In fact, available literature shows that there are few studies and reports on cigar aging. On the one hand, cigar aging is a critical process to improve cigar quality, thus most cigar manufacturers do not publicize the aging technology. On the other hand, the mechanism of cigar aging is complex, so there is insufficient support for the evaluation of cigar aging technology.

Studies on the mechanism of cigar production technology often focus on the fermentation and air-drying stages, since these two processes exhibit observable physical and chemical changes in tobacco leaves, especially the color and elasticity of tobacco leaves (Liu et al., 2021; Ren et al., 2023). Researchers typically investigated alterations in major chemical components such as aroma compounds, nicotine content, sugar content, as well as the succession patterns of microbial communities during these stages. Additionally, incorporating media into these stages serves as an important approach to enhance the quality of cigar tobacco leaves. Zong et al. (2023) explored the effects of adding cocoa as the fermentation medium on the quality of eggplant core cigar leaves. Lu et al. (2023) conducted a study on the effects of exogenous melatonin on lipid peroxidation level of cigar tobacco leaves during air-curing stage.

However, compared to fermentation and drying stages, there is currently few studies on the exploration of new aging media and effects of aging media on physicochemical properties of cigar. Therefore, to further expand the aging methods of cigar and to clarify the change laws of chemical properties of cigar during aging process, this paper studied the effects of aging with different media on the quality of cigar. Firstly, sensory evaluation was conducted as the main evaluation index for screening new aging media. Then, changes in chemical composition and succession of microbial community during the aging process of cigar coupled with characteristic aging media were analyzed. This study aims to broaden the aging methods and new approaches to improve the fragrance and quality of cigars.

## 2 Materials and methods

### 2.1 Experiments design

The aging experiments were carried out using GreatWall (Lansheng No. 3) as a representative cigar sample, which were produced by the GreatWall Cigar Factory (De-yang, China). The cigar samples were frozen at −20°C for 2 days after rolling to prevent from insects. Coffee formula, cocoa formula, rose formula, liquor formula I, liquor formula II, and red wine formula were provided by Jiangnan University. Chemical reagents used in this study were of analytical grade unless otherwise stated.

**FIGURE 1:**
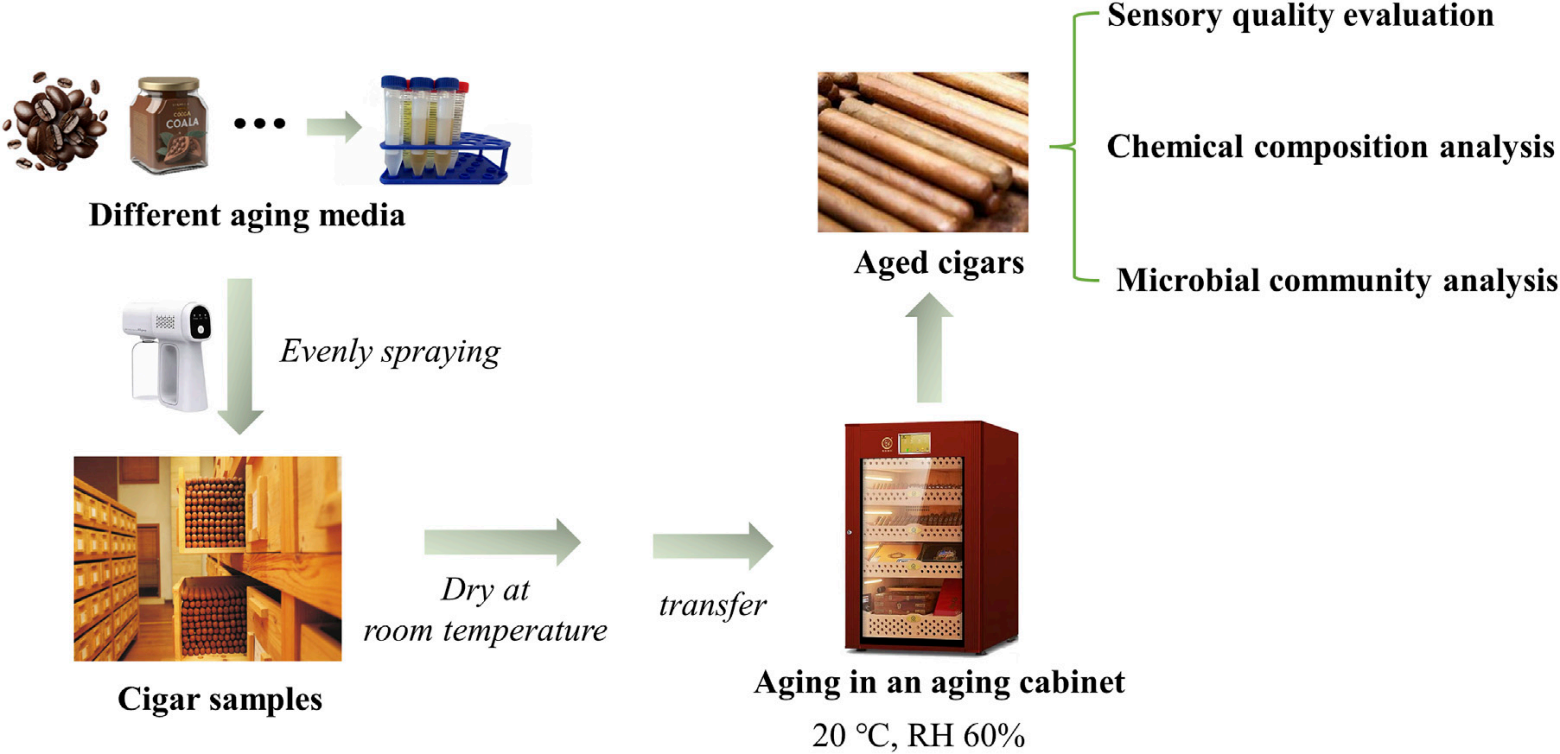
The procedure of the aging experiment.

Detailed steps of aging process are shown in Figure 1. In a typical aging experiment, 50 mL of coffee formula was sprayed evenly over the surface of 50 cigars. The cigars were then dried at room temperature until no ruing water on the surface, and transferred to an aging cabinet (Bulldog, VC528) at a temperature of 20°C and relative humidity of 60%. The cigars were randomly sampled during the aging process for sensory evaluation, chemical composition and microbial community structure analysis. Two control groups were established for comparison, *i.e.*, blank aging and medium aging followed by transfer to a blank environment. In addition, the coffee formula was successively replaced by cocoa formula, rose formula, liquor formula I, liquor formula II and red wine formula. The sampling inspection was set up as shown in Table 1 (using the coffee group and cocoa group as examples).

**TABLE 1:** Table of sample inspection.

Sample names	Aging process
W-30	Aging for 30 days in blank group
C-30	Aging with coffee formula for 30 days
C30-W30	Aging with coffee formula for 30 days, then aging for 30 days in blank group
C-60	Aging with coffee formula for 60 days
C60-W30	Aging with coffee formula for 60 days, then aging for 30 days in blank group
C-90	Aging with coffee formula for 90 days
K-30	Aging with cocoa formula for 30 days
K30-W30	Aging with cocoa formula for 30 days, then aging for 30 days in blank group
K-60	Aging with cocoa formula for 60 days
K60-W30	Aging with cocoa formula for 60 days, then aging for 30 days in blank group
K90	Aging with cocoa formula for 90 days

### 2.2 Sensory quality evaluation

The sensory qualities of aged cigars were evaluated by a smoke evaluation team, consisting of five smoke evaluation experts. The sensory quality evaluation standard of the Great Wall Cigar Factory was adopted to determine scores (Li et al., 2022).

### 2.3 Analysis of starch

The determination of starch content in cigars was based on a trade standard in China through iodine-chromogenesis (Chen et al., 2023). In detail, 0.2 g of cigar powder (through a 40 mesh sieve) was accurately weighed and placed in a triangular flask. Then, 25 mL of 80% ethanol-sodium chloride saturated solution was added to the triangular flask and shaken at 50°C for 20 min. The sediment was obtained by filtration, and 10 mL of 40% perchloric acid solution was added to the filter residue, which was then shaken at room temperature for 25 min. The filter liquid was collected and distilled water was added until the liquid volume was 100 mL. Then 10 mL of the liquid and 1 mL of potassium iodide solution were mixed, and the absorbance of the mixture was measured at 600 nm using a UV-visible spectrophotometer. Each sample was tested three times in parallel.

### 2.4 Analysis of amino acid

0.1 g of cigar powder and 8 mL of hydrochloric acid (6 M) were added to a hydrolysis tub filled with nitrogen for 3 min to keep the liquid in a slightly boiling state. The hydrolysis tube was placed in an oven at 120°C for 22 h. The hydrolysate was then mixed with 4.8 mL of NaOH (10 M) with distilled water until the total volume was and 25 mL. The clarified filtrate was obtained after filtration through double layer filter paper, centrifugation (15,000 rpm for 30 min) and filtration through 0.22 μm water membrane in turn.

Amino acid detection was performed by HPLC, which was coupled with a Hypersil ODS C18 column (4.6 mm × 250 mm). The column temperature was 40°C and the flow rate was 1.0 mL min^−1^. Two detectors were used: a UV detector (338 nm; 22.5 min 262 nm) and a fluorescence detector (excitation wavelength of 340 nm and emission wavelength of 450 nm). Th mobile phase A was 0.03 M sodium acetate containing 0.5% of tetrahydrofuran (pH 7.20 ± 0.05), while the mobile phase B was the mixture of acetonitrile and methanol (V:V = 4:6, pH 7.20 ± 0.05).

Additionally, analysis of non-volatile organic acids and aromatic compounds, as well as the DNA extraction and sequencing were performed according to our previous study (Hu et al., 2022).

## 3 Results and discussion

### 3.1 Sensory evaluation

**FIGURE 2:**
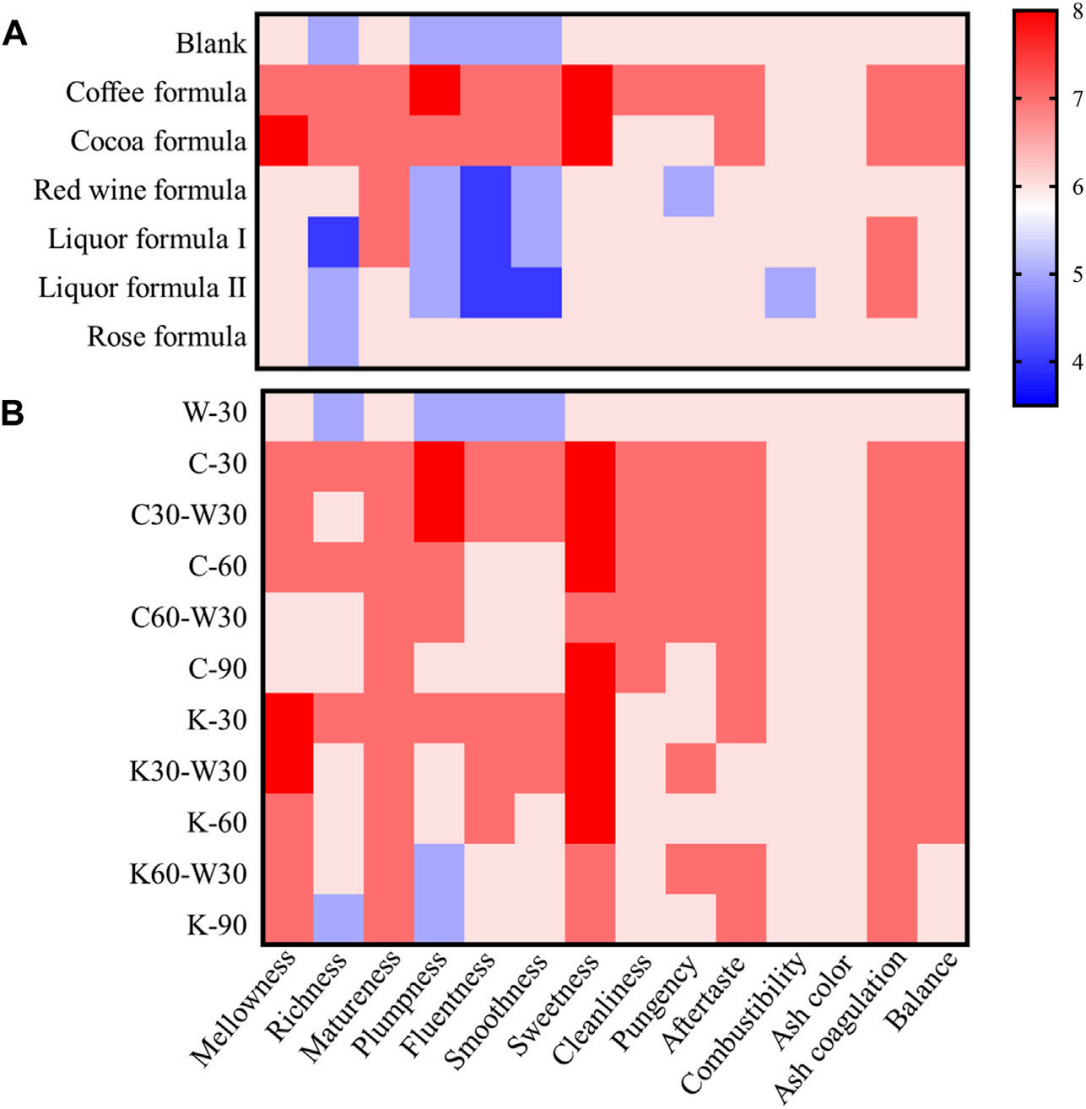
The sensory quality of cigars. **(A)** the effect of different aging media on sensory quality, **(B)** the effect of different aging time on sensory quality.

In this study, a comparative analysis of sensory quality was carried out on cigars aged with the six media and those in the blank control group, as shown in Table 2 and Figure 2A. It can be seen that the cigar of blank control group exhibited honey and nut aromas as its main flavor, complemented by burnt sweetness, wood and bean aromas. The aroma was mellow, the smoke was smooth, and the aftertaste was clean. However, its flavor richness was mediocre. Therefore, it is necessary to explore available aging media to improve the quality of cigar.

**TABLE 2:** Sensory quality evaluation of cigars.

Sample	Sensory quality
Blank	Honey-sweet and nutty aromas were found in this sample, which performed well in terms of smoothness, fluidity and aftertaste. However, a relatively low level of flavor richness was noted
Coffee formula	The plumpness and concentration of the smoke as well as the aftertaste were significantly improved. Nutty, caramel, and baked flavor were detected
Cocoa formula	The mellowness and sweetness were improved, and the permeability of the smoke was upgraded. Besides, a relatively fragrant flavor was found
Red wine formula	This sample showed increased sweetness and obvious red wine flavor, but the smoothness and cleanliness of smoke were destroyed
Liquor formula I	The pungency of the smoke was slightly reduced. However, the cleanliness and smoothness of smoke decreased
Liquor formula II	The pungency of smoke decreased slightly. However, the cleanliness and smoothness of smoke decreased
Rose formula	The prominent floral and honey-sweet notes were captured, but the nutty scent was masked. The smoothness and fluentness, as well as aftertaste were also improved. However, the original fragrance characteristics of the cigar were damaged and the coordination was poor

As illustrated in Table 2; Figure 2A, the sensory quality of cigar was modified after aging with media for 30 days. It can be seen that among the six media, coffee formula and cocoa formula showed the most positive effect on the sensory quality of cigar. The plumpness and concentration of smoke aged with coffee formula were significantly improved, and the aromas of burnt sweet, roasted and nut, as well as the aftertaste were enriched. In addition, the mellowness and sweetness of cigars aged with cocoa formula were improved obviously, as was the permeability of smoke. By contrast, aging with liquor formula I, liquor formula II, red wine formula, or rose formula had no significant effect on the sensory quality of cigar. After aged with red wine formula, the cigars showed increased sweetness and obvious red wine flavor, but the reduced smoothness and cleanliness of smoke were found. Cigars aged with liquor formula I or liquor formula II showed the improvement in fragrance, but a deterioration in smoke smoothness. Additionally, rose formula significantly improved the aroma richness of cigar, and endowed cigar with aromas of flower and honey. However, the original aroma characteristics of the cigar were damaged and its coordination was poor. In fact, different cigars displayed different styles and main aromas. For example, the Lansheng No. 3 selected for this research showed the nutty aroma as main flavor, so the coffee and cocoa formulations matched the style of Lansheng No. 3. Therefore, although the rose formula is not suitable for the aging of Lansheng No. 3, it can be considered for other brands of cigar with floral fragrance, such as Changcheng Red 132 (China)^1^.

It can be concluded that coffee formula and cocoa formula showed a remarkable improvement in the sensory quality of cigar. Therefore, this study continued to analyze the effects of different aging time on the sensory quality of cigars with coffee formula or cocoa formula.

As shown in Figure 2B, with the extension of aging time, the sensory quality of cigar decreased, which exhibited the best performance at 30 days. For the coffee group, after aged for 30 days, the flavor richness, smoke fullness and sweetness of cigar were significantly improved compared with the blank group (W-30). The performance of C-60 on smoke characteristics (including plumpness, fluentness, and smoothness) was poorer than C-30. After aged for 90 days, the mellowness and aroma richness of cigar decreased, and the irritation increased. Whereas, C-90 still possessed a higher score than W-30. As for the cocoa group, the improvement of mellowness, aroma richness and sweetness were observed in K-30. The decrease of mellowness was found as the aging time increased. This is due to that the long-term aging with cocoa resulted in the cigar with heavy residue and broken smoothness. The above results showed that the quality of cigar aged with coffee formula or cocoa formula was improved obviously, and the appropriate aging period was 30 days.

**FIGURE 3:**
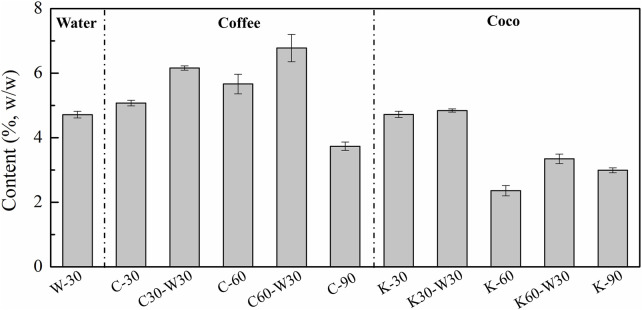
The starch content of cigars aged with different media.

Considering that consumers tend to store cigars for a period of time before smoking, this study explored the sensory quality degradation of cigars removed from a medium environment and stored in a conventional aging condition. As shown in Figure 3, C30-W30 and C-30 possessed higher sensory quality scores than C60, while C60-W30 and C-60 had similar and higher sensory quality scores than C-90. Similar phenomena were also found in the cocoa group. It suggested that storing cigars in a conventional environment for 30 days after aged with media has little effect on the sensory quality of cigars, and consumers do not need to worry about quality deterioration during storage. In general, the sensory quality of cigars was significantly improved after aged with coffee formula or cocoa formula for 30 days.

### 3.2 Starch analysis

Figure 3 shows the effect of aging media on the starch content. It can be seen that cigars aged with coffee formula had a relatively higher content of starch, which were all above 5%, except for C-90. By contrast, cocoa group exhibited lower content of starch than coffee group.

Significance analysis of starch content in coffee- and cocoa-groups was carried out to further analyze the effects of aging time and methods on starch content. As shown in Supplementary Figure S1, a declining trend of starch content in cigar was detected with the extension of aging time. In the coffee group, there was no significant effect on the starch content of C-30 and C-60, which showed a higher level than that in C-90. In the cocoa group, the starch content of K-60 and K-90 was lower than that of K-30. As shown in Supplementary Figure S2, the starch contents in medium-combined with blank-aging group were higher than those in medium group. The starch content of C60-W30 was obviously higher than that of C-90. A similar phenomenon was observed in cocoa group (K-60 vs*.* K30-W30 and K-90 vs*.* K60-W30). It can be concluded that the addition of coffee or cocoa media has little effect on the starch content of cigar, while the prolongation of aging time was negatively correlated with starch content. It may be related to the amylase or amylase-producing microbial activities (Jin et al., 2022; Ning et al., 2023). Therefore, more attention should be paid to the content and activity of amylase in cigar.

### 3.3 Amino acid analysis

**FIGURE 4:**
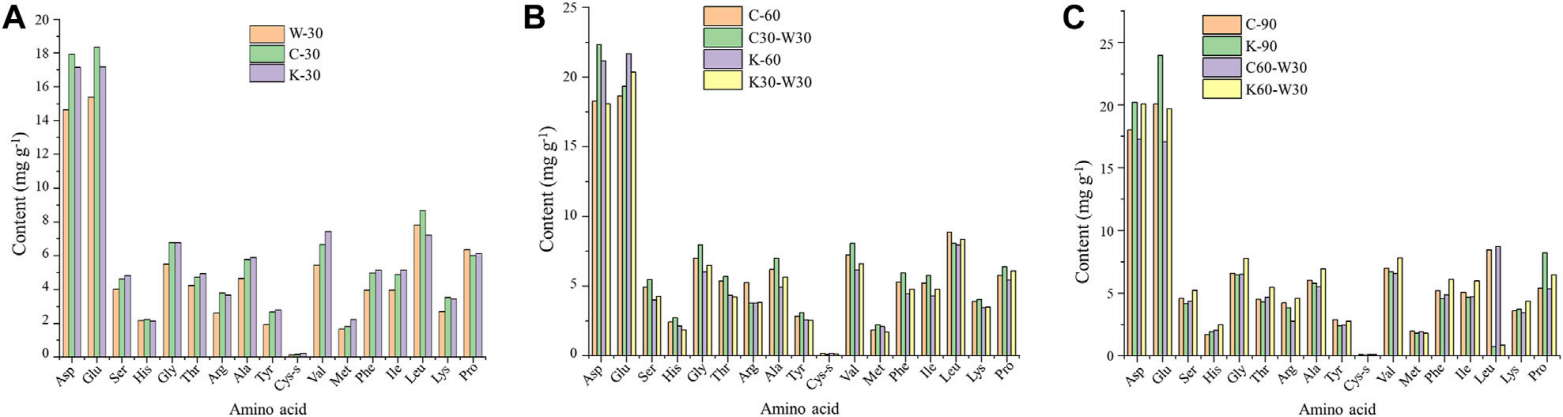
Effects of aging with coffee or cocoa media on amino acid content of cigars. **(A)** aging for 30 days **(B)** aging for 60 d **(C)** aging for 90 days.

The amino acid composition and content of cigar were analyzed by HPLC. As shown in Figure 4A, compared with W-30, the contents of Asp, Glu, Ser, Gly, Thr, Arg, Ala, Tyr, Val, Met, Phe, Ile, Leu, Lys, Pro were higher in C-30 and K-30. Particularly, aging with coffee or cocoa exhibited an obvious effect on the contents of Asp and Glu, since the Asp content increased by 22.40% and 17.14%, and the Glu content increased by 19.32% and 11.84%, respectively. Besides, the total amino acid content of C-30 and K-30 increased from 87.08 mg g^−1^ (W-30 in blank group) to 103.52 and 102.28 mg g^−1^, respectively, indicating that the two aging media could significantly increase the amino acid content of cigar.

From Figure 4B, C30-W30 had higher content of amino acid than C-60, especially Asp (increased by 22.39%). However, there was no significant difference in amino acid content between K30-W30 and K-60. Compared with coffee group, the contents of Asp and Glu in cocoa group were higher, while those of other amino acids were lower, indicating that the effect of aging with coffee formula on amino acid content of cigar was obvious than that of aging with c cocoa formula. When aging for 90 days, the contents of Asp, Glu and Pro in cocoa group were higher than those in coffee group, while the content of other amino acids was lower (Figure 4C). Besides, C-90 showed the relatively high content of virous amino acids than C60-W30, and the contents of Asp, Glu, Met and Pro in K-90 were higher than that in K60-W30. The total amino acid content of C-60, C30-W30, K-60 and K30-W30 was 108.99, 117.76, 104.48, and 103.05 mg g^−1^, respectively, while that of C-90, C60-W30, K-90 and K60-W30 was 105.36, 98.37, 103.48, and 108.48 mg g^−1^, respectively. It can be seen that aging in traditional environment after removed from the media environment showed an insignificant effect on the amino acid content of cigar.

For the coffee group, the contents of Glu, Try and Met increased as aging proceeded, while the content of Pro decreased. The content of other amino acids increased firstly and then decreased, which reached a highest level at 60 days. For the cocoa group, the contents of Asp and Leu reached the maximum value at 60 days, and the contents of Glu and Pro increased with the increasing of aging time and reached the maximum value at 90 days. In addition, the extension of aging time had no significant effect on the amino acid contents of cigar.

Amino acids were important flavor components of cigar, which would be pyrolyzed to produce pyrrole, indole or benzoic acid and other flavor components to enrich the flavor of cigar during the burning process (Peng et al., 2021; Jia et al., 2023). Therefore, in this study, the addition of two aging media can significantly increase the amino acid content of cigar, thus improving the aroma richness of cigar.

### 3.4 Organic acid analysis

**FIGURE 5:**
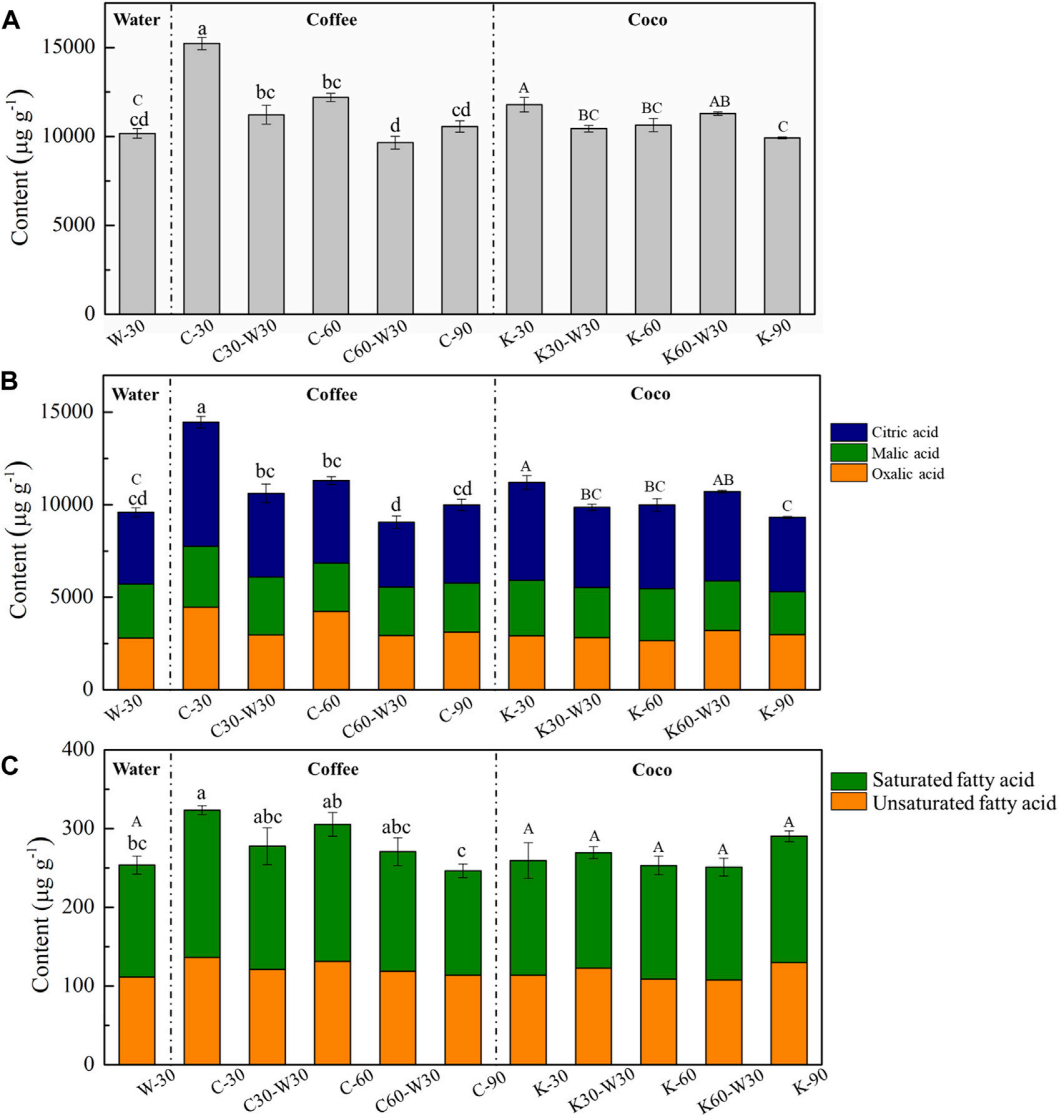
The contents of organic acids in cigars. Note: Different lowercase letters indicate that the difference of samples in water and coffee groups is statistically significant at *p* < 0.05 level. Different capital letters indicate that the difference between samples in water and cocoa groups is statistically significant at *p* < 0.05 level (same means for the following figures and tables). **(A)** is the content of non-volatile organic acids, **(B)** is the contents of small molecular organic acids, **(C)** is the fatty acids contents.

Non-volatile organic acids play an essential role in the sensory quality of tobacco, since they are the important intermediates of tricarboxylic acid cycle (Guan et al., 2023). Besides, some organic acids and their derivatives contributed significantly to the flavor and taste of tobacco (Li et al., 2023). The effect of aging with coffee or cocoa formula on the content of non-volatile organic acids in cigars was investigated. As depicted in Figure 5A, C-30 (15,221.65 μg g^−1^) and K-30 (11,786.01 μg g^−1^) showed the obviously higher content of non-volatile organic acid than W-30 (10,174.63 μg g^−1^), indicating that coffee and cocoa formula introduced rich non-volatile organic acids into cigar (Greno et al., 2022; Febrianto and Zhu, 2023). With the extension of aging time, the content of non-volatile organic acids in the coffee and cocoa groups decreased to 10,566.82 and 9,920.39 μg g^−1^ at 90 days, respectively, which were similar to that of W-30. For the coffee group, reduced non-volatile organic acid content was found in medium aging followed by blank aging group. For the cocoa group, medium aging followed by blank aging has little effect on the content of non-volatile organic acids of cigars. Thus, cigar aging with coffee or cocoa formula could increase the content of non-volatile organic acids, so as to regulate the acidity and alkalinity of smoke.

Citric acid, malic acid and oxalic acid are the main non-volatile organic acids in cigars, whose contents ranged from 3,499.47 to 6,722.16 μg g^−1^, 2,318.31–3,281.68 μg g^−1^ and 2,651.21–4,464.84 μg g^−1^, respectively (Figure 5B). These three organic acids accounted for more than 95% of non-volatile organic acids. The content of citric acid showed a decreasing trend as aging with coffee or cocoa formula as aging proceeded, which finally reached 4,224.25 and 4,021.03 μg g^−1^, respectively. Compared to that of C-30, the citric acid content of C30-W30 dropped by about 30%. Malic acid could reduce the acidity of smoke and affect the characteristics of smoke, which is conducive to high-nicotine plants (Lu et al., 2021). In this study, the content of malic acid in coffee or cocoa group was higher than that in blank aging group at 30 days. In addition, the malic acid content in cocoa group showed a decreasing trend as aging proceeded (from 2,994.91–2,318.31 μg g^−1^), while the malic acid content in coffee group first decreased and then increased. Besides, the oxalic acid content of C-30 was significantly higher than that of W-30 and K-30. As the aging time increased, oxalic acid content in coffee group decreased, while that in cocoa group first decreased and then increased, reaching the highest level at 90 days.

Generally, higher fatty acids have no obviously direct effect on tobacco smoke and aroma, but they can adjust the pH and increase the mellowness of smoke, which play a balancing role in cigars indirectly (Yang et al., 2017). It is believed that saturated higher fatty acids can enhance the aroma richness and mellowness of smoke, while excessive unsaturated fatty acids would increase the irritation and roughness of smoke (Li et al., 2020). As illustrated in Figure 5C, the content of saturated fatty acids in C-30 was significantly higher than that in W-30, while cocoa aging had no significant effect on the content of saturated fatty acids in cigars. Besides, the content of unsaturated fatty acids decreased significantly from 30 days to 90 days. However, no significant difference in the content of unsaturated fatty acids was found in K-30 and K-90. It demonstrated that compared with cocoa group, extending the aging time in coffee group can reduce the content of unsaturated fatty acids in cigar, thus reducing the irritation and improving the sensory quality of cigar.

### 3.5 Aroma component analysis

**FIGURE 6:**
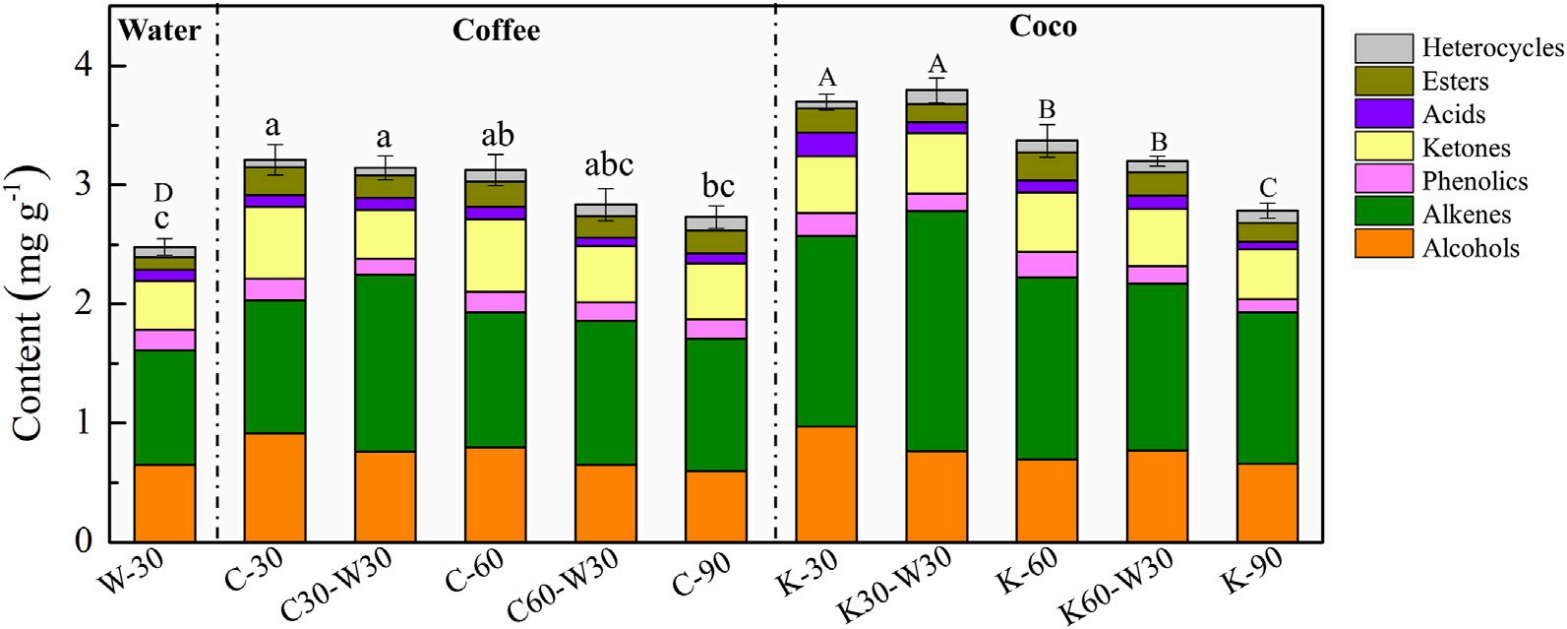
Effects of aging media on the content of aroma component in cigars.

Figure 6 shows the effect of aging media on the content of aroma components in cigar. It can be seen that aging with coffee or cocoa formula significantly increased the content of aroma components which increased from 2.48 mg g^−1^ (W-30) to 3.21 mg g^−1^ (C-30) and 3.70 mg g^−1^ (K-30), respectively. It indicated that the cigars were enriched with flavors from coffee and cocoa formulas. According to previous reports, abundant volatile and non-volatile aroma compounds were found in coffee and cocoa (Carcea et al., 2023; Ullrich et al., 2023). With the extension of aging time, the content of aroma components in coffee and cocoa group showed a decreasing trend and reached the lowest level at 90 days, because aroma components in cigar introduced by coffee and cocoa would be continuously lost during aging process. In addition, there was no significant difference between C-30 and C30-W30, C-60 and C60-W30, K-30 and K30-W30, as well as K60 and K60-30 in the content of aroma components, indicating that the aroma components in cigar would not be significantly lost when placed in the conventional aging environment. It is consistent with the sensory evaluation results in Secion 3.1.

Except for the total content of aroma components, this study also analyzed the change rule of different types of aroma substances. It can be seen that the aroma components of alcohols, alkenes, ketones and esters were enriched with the addition of coffee formula, while cocoa aging significantly improved the contents of alcohols, alkenes, acids and esters. The increasement of ester compounds reached by 126.27% and 96.81% in coffee and cocoa groups, respectively, which is related to the abundant ester compounds in coffee and cocoa (Yi et al., 2015; Quelal et al., 2023). Besides, the changing trend of various aroma substances was similar to that of the total aroma components, which showed a downward trend. Therefore, according to the variation trend of aroma components, aging with coffee or cocoa medium for 30 days was beneficial in increasing the content of flavor-causing ingredients and enriching the flavor of cigar.

### 3.6 Microbial community analysis

The microbiota was categorized into fungi and bacteria populations. As shown in Table 3 and Supplementary Figure S3, rarefaction curves and Good’ coverage percentage (higher than 99.97%) indicated that the sequencing depths were sufficient to reflect the microbial composition of cigars.

**TABLE 3:** Richness and diversity of microbial communities of cigars.

Samples	Shannon index		Simpson index		Ace index		Chao index		Coverage (%)	
	Bacteria	Fungi	Bacteria	Fungi	Bacteria	Fungi	Bacteria	Fungi	Bacteria	Fungi
W-30	1.68	2.16	0.32	0.22	103.42	70.61	100.58	70.33	99.99	100.00
C-30	0.24	2.26	0.93	0.16	84.45	91.99	82.65	91.38	99.98	99.99
C30-W30	1.29	1.52	0.38	0.32	120.46	53.00	115.25	53.00	99.98	100.00
C-60	0.68	2.10	0.79	0.29	121.85	165.08	122.00	164.67	99.98	99.99
C60-W30	0.90	2.10	0.65	0.25	132.57	89.70	128.96	89.14	99.98	99.99
C-90	0.93	1.52	0.59	0.41	126.09	57.27	125.06	57.00	99.98	100.00
K-30	0.50	2.01	0.79	0.20	115.43	58.92	126.67	58.00	99.97	99.99
K30-W30	0.28	1.73	0.92	0.27	55.94	54.26	56.60	54.00	99.99	100.00
K-60	0.22	2.07	0.94	0.20	139.69	50.20	82.14	50.00	99.97	100.00
K60-W30	0.39	0.86	0.86	0.63	72.64	20.00	72.33	20.00	99.98	100.00
K-90	0.65	1.86	0.79	0.25	91.06	53.89	90.50	53.33	99.99	100.00

Alpha diversity metrics, including Chao, Shannon, Simpson, and Ace, were generated to reflect the microbial community diversity and richness of cigar samples (Table 3). Results showed that bacterial Chao index of coffee group was basically higher than that of blank group, while that of cocoa group was similar to that of blank group. In the coffee group, the Chao index of bacteria increased during the first 60 days, which changed insignificantly in 60–90 days. Moreover, the effect of aging in the blank environment was not obvious in coffee group (C30-W30 vs. C-60, C60-W30 vs. C-90). The Chao index showed a decreasing trend as increasing aging time in cocoa group. In addition, the variation trend of Shannon index is consistent with that of Chao index, but Shannon index of the bacterial diversity with two aging methods was lower than that of blank group. These above results indicated that aging with coffee medium could improve the bacterial diversity of cigar, while aging with cocoa medium exhibited no significant effect on the bacterial diversity of cigar.

As shown in Table 3, considerable differences were observed in the Chao index of fungal diversity between coffee group and cocoa group. In coffee group, the Chao index increased continuously during the first 60 days, which also increased significantly during the following 30 days of blank aging (C60-W30). These results indicated that aging with coffee medium could improve the diversity of fungi, while aging with cocoa had no significant effect on the diversity of fungi on cigar surface.

**FIGURE 7:**
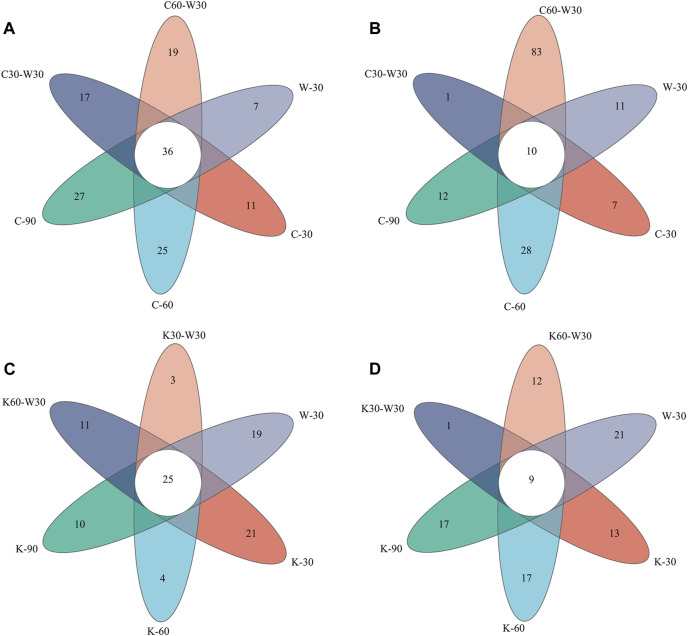
Venn diagram of OTU distribution. **(A)** shows the bacterial communities of cigar in coffee group. **(B)** shows the fungal communities of cigar in coffee group. **(C)** shows the bacterial communities of cigar in cocoa group. **(D)** shows the fungal communities of cigar in cocoa group.

Figure 7 shows the Venn diagrams of OTU level in coffee and cocoa group. There were 142 and 93 bacterial OTUs in the coffee and cocoa groups, of which 36 and 25 were common, respectively. It showed that the unique bacterial group played a role in the media-aging process. The number of unique OTU of most cigar samples increased significantly compared to that of blank group, and the highest number of unique OTU was 27 (C-90). It indicated that cigars aged with coffee formula showed the obvious superiority in the number of unique bacteria. After 30 days of blank aging, the total number of OTU remained stable, demonstrating that aging with coffee could not only improve the bacterial diversity, but also keep cigar with stable bacterial diversity in the subsequent storage period. As shown in Figure 7B, the number of common fungal OTU on the coffee-aged cigar was 10, and the three samples C-30 through C-90 had 7, 28, and 12 unique OTUs, indicating that the unique functional fungi in each stage is closely related to the aging process, with an obvious succession phenomenon at play. These results indicated that aging with coffee formula for 60 days could significantly promote the increase of the unique fungal community on cigar surface.

As shown in Figure 7C, the number of unique OTU of cigars aged with cocoa decreased significantly compared with that of blank group. K-30 possessed the highest number of unique OTU (21) among all samples, which decreased as the aging process extended to 60 or 90 days. The results showed that aging with cocoa had no obvious advantage in increasing the number of bacterial OTU in cigar. In addition, the number of common fungal OTU in cocoa group was 9, and the unique OTU of cigar samples was relatively small, indicating that aging with cocoa formula had no significant effect on fungal diversity of cigar.

**FIGURE 8:**
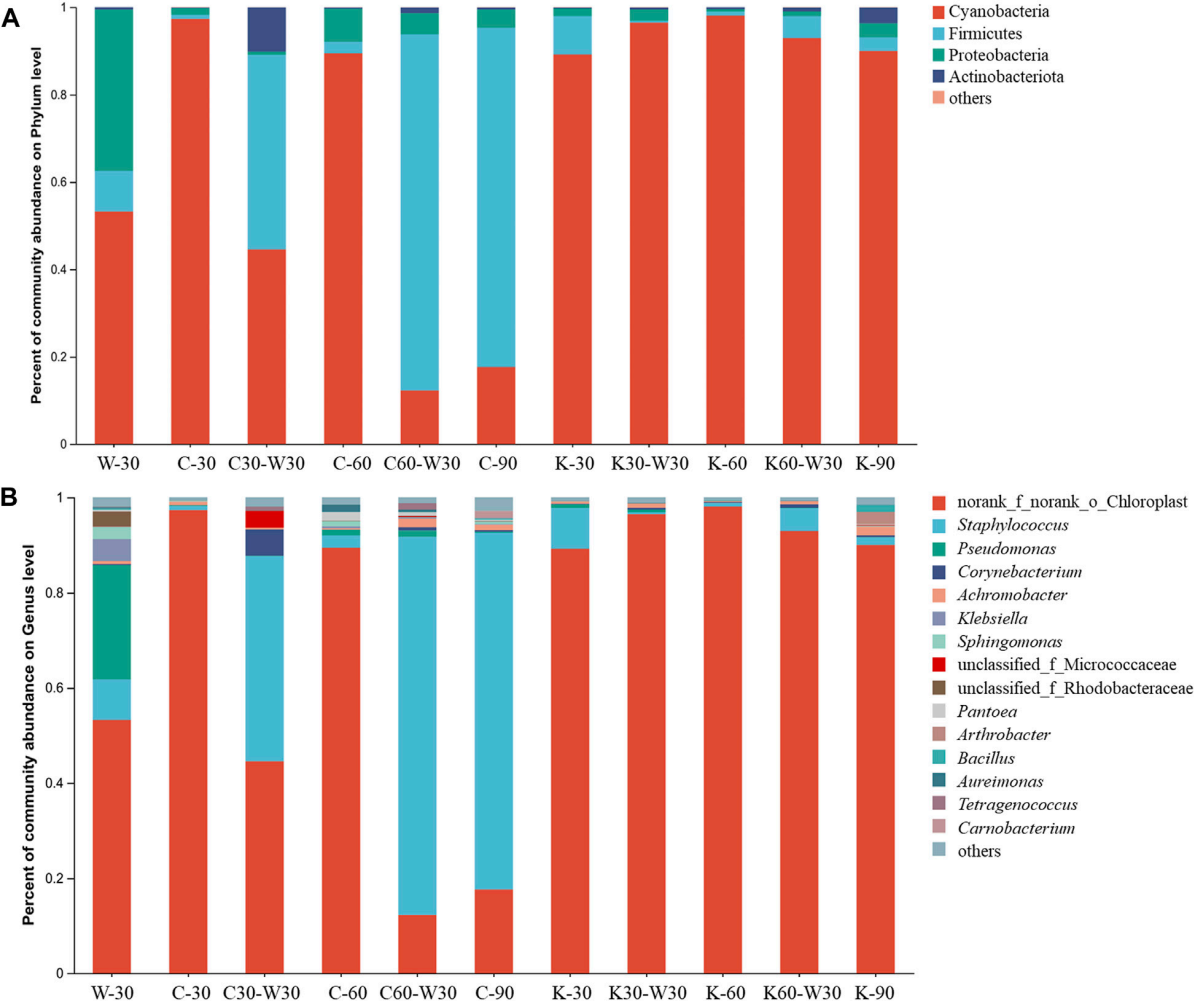
Bacterial community structure of cigars. **(A)** the phylum level, **(B)** the genus level.

As shown in Figure 8A, the bacterial community in coffee and cocoa groups mainly consisted of four phyla, which were Cyanobacteria, Firmicutes, Proteobacteria and Actinobacteriota. Among them, Cyanobacteria and Firmicutes were the dominant communities, whose relative abundance was more than 85%. In the blank group, most bacteria (>90%), were members of the phylum Cyanobacteria and Proteobacteria. After aging with coffee, the relative abundance of Cyanobacteria in cigar decreased as increasing the aging time. Compared with W-30, C30-W30 showed a decreased relative abundance of Cyanobacteria and an increased relative abundance of Firmicutes. In the cocoa group, Cyanobacteria were the dominant bacteria, and the relative abundance was above 90%.

As shown in Figure 8B, the major group bacteria of cigar could be categorized into norank_f_norank_o_choroplast, *Staphylococcus*, *Pseudomonas* and *Corynebacterium*. The bacterial community in W-30 was dominated by norank_f_norank_o_choroplast and *Pseudomonas*. By contrast, C-30 and K-30 showed the relatively higher abundance of norank_f_norank_o_choroplast. In coffee group, the relative abundance of norank_f_norank_o_choroplast decreased with the increase of aging time, and *Staphylococcus* gradually took the dominant position. The relative abundance of norank_f_norank_o_choroplast and *Staphylococcus* in C30-W30 were lower than those in C-60. For cocoa group, norank_f_norank_o_choroplast always occupied the dominant position, and its relative abundance did not change significantly with aging time and aging method. These results indicated that the addition of coffee medium was beneficial for the enrichment of *Staphylococcus* in cigars at the later stage of aging. Compared with blank aging, coffee and cocoa aging showed significant effects on the bacterial community of cigar. Besides, the aging time had little effect on the bacterial community at the phylum level in cocoa group, but aging with coffee could accelerate the succession rate of bacterial community and significantly change the succession rule of microbial community.

**FIGURE 9:**
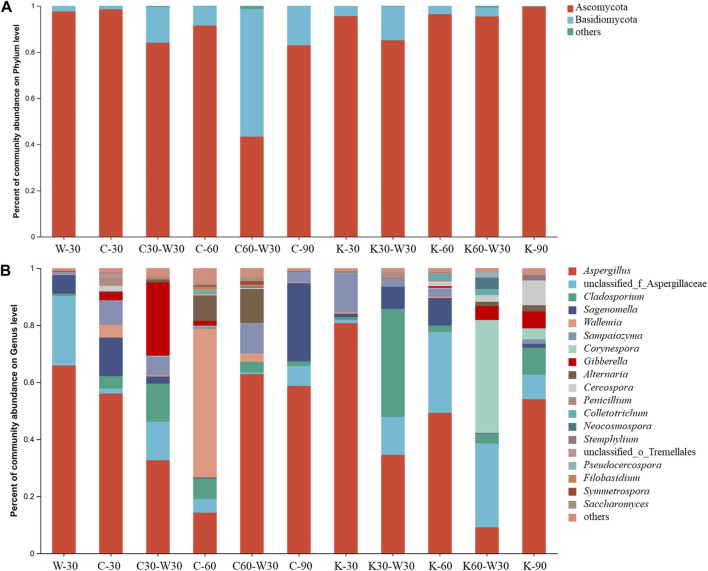
Fungal community structure of cigars. **(A)** the phylum level, **(B)** the genus level.

As shown in Figure 9A, the fungal community in cigars mainly consisted of two phyla (Ascomycota and Basidiomycota). During the aging process, Ascomycetes were the dominant fungi, and their relative abundance accounted for more than 80%. For W-30, Ascomycota was the dominant fungi with relative abundance of about 95%. For coffee group, the relative abundance of Ascomycetes decreased as aging proceeded. The relative abundance of Ascomycetes decreased and that of Basidiomycetes increased in coffee combined with blank aging group. For cocoa group, Ascomycota was the dominant fungi, and its relative abundance did not change significantly as aging proceeded, further indicating that aging with cocoa had little effect on the fungal community in cigar at the phylum level.

As shown in Figure 9B, *Aspergillus*, unclassified_f_Aspergillaceae, *Cladosporium*, *Sagenomella*, *Wallemia*, *Sampaiozyma* and *Corynespora* were the six main dominate fungal genera. With the addition of aging medium, the relative abundance of *Sagenomella* in coffee group (C-30) and *Sampaiozyma* in cocoa group (K-30) increased. The relative abundance of *Wallemia* increased significantly (more than 50%) at 60 days of coffee group, and the relative abundance of *Aspergillus* reached 50%–60% at 30 and 90 days, showing a similar value to W-30. For cocoa group, K-30 and K-90 possessed the dominant fungi of *Aspergillus*. Unclassified_f_Aspergillaceae, *Cladosporium*, and *Corynespora* alternated as the dominant fungi during the aging process. Although the addition of coffee and cocoa medium has no significant effect on the fungal community at the genus level, *Aspergillus* was always the dominant fungi. Considering that *Aspergillus* was known to be able to cause mould (Li et al., 2023), ambient temperature and humidity should be strictly controlled during the aging process to reduce the risk of mould.

## 4 Conclusion

In summary, two novel aging media were prepared and their effects on the sensory quality, chemical composition and microbial community structure of cigar were investigated. The systematic studies manifested that aging cigar with coffee or cocoa formula could significantly optimize the sensory quality of cigar, including the aroma richness, smoke characteristics, and aftertaste. Moreover, the contents of amino acids, non-volatile organic acids and aroma components of cigar were improved with the addition of media. The decreased starch content was detected. Besides, coffee formula was proved to increase the diversity of the fungal and bacterial community on cigar surface, and changed the structure of bacterial community. The findings in this study not only display two novel aging media, but also reveal the influence of aging media on cigar quality multidimensionally for the first time. Additionally, effective strategy for developing new technologies of enhancing cigar quality was proposed.

## Data Availability

The datasets presented in this study can be found in online repositories. The names of the repository/repositories and accession number(s) can be found below: https://www.ncbi.nlm.nih.gov/sra/PRJNA1016040 and https://www.ncbi.nlm.nih.gov/sra/PRJNA1016060.

## Footnotes

1 China Tobacco Sichuan Industrial Co. Ltd. (2023) Production http://10.165.1.194/portal/product/6901028146005
