# Supplementary material for: Sensory attributes, chemical and microbiological properties of cigars aged with different media
Source: Front Bioeng Biotechnol. 2023 Oct 24;11:1294667. doi: 10.3389/fbioe.2023.1294667 (PMC10628719; doi:10.3389/fbioe.2023.1294667)
Supplement: Supplementary file 1 [file DataSheet1.docx]

Supplementary Material

**List of Contents**

**Supplementary Figure S1.** Effect of aging time on starch content of cigar. (a) Coffee-group, (b) Cocoa-group

**Supplementary Figure S2.** Effect of medium-aging combined with blank-aging on starch content of cigar. (a) Coffee-group, (b) Cocoa-group

**Supplementary Figure S3.** Rarefaction curves. (a) shows the bacterial communities of cigar in coffee group. (b) shows the fungal communities of cigar in coffee group. (c) shows the bacterial communities of cigar in cocoa group. (d) shows the fungal communities of cigar in cocoa group.

**
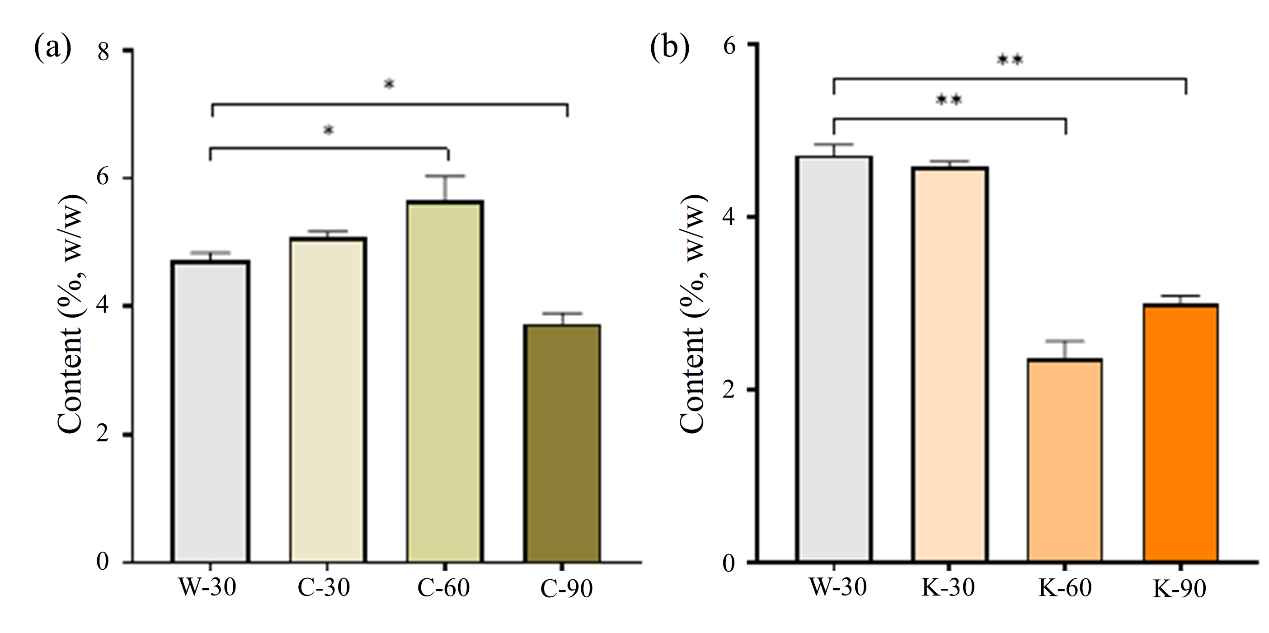
**

**Supplementary Figure S1. Effect of aging time on starch content of cigar. (a) Coffee-group, (b) Cocoa-group.**

**
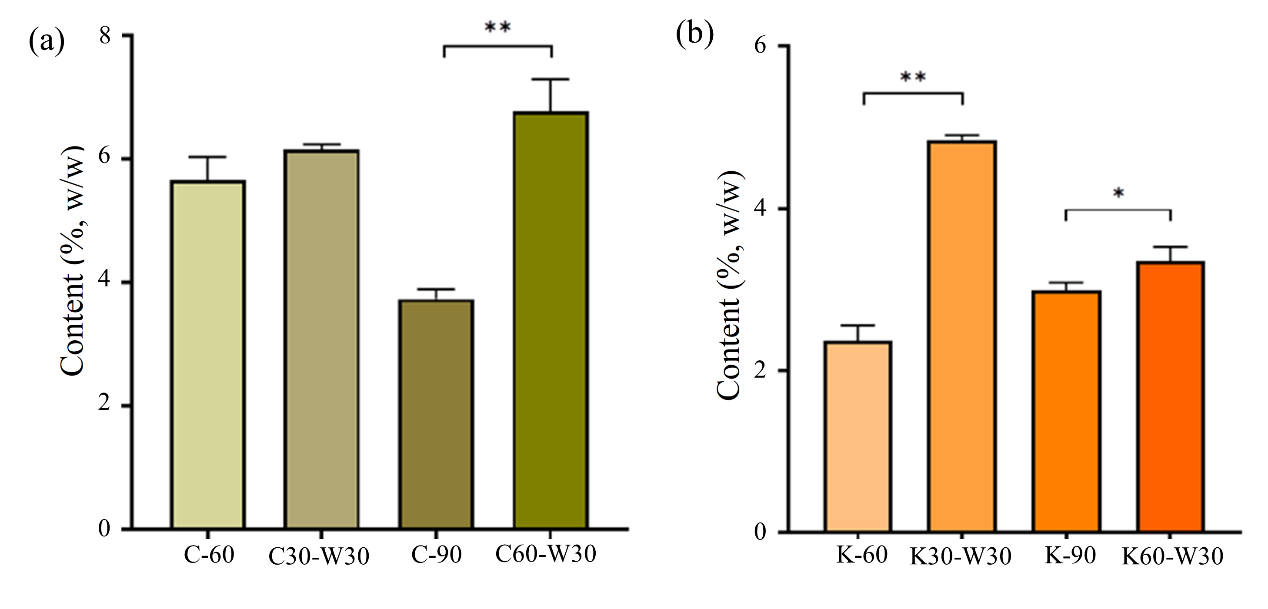
**

**Supplementary Figure S2. Effect of medium-aging combined with blank-aging on starch content of cigar. (a) Coffee-group, (b) Cocoa-group**

**
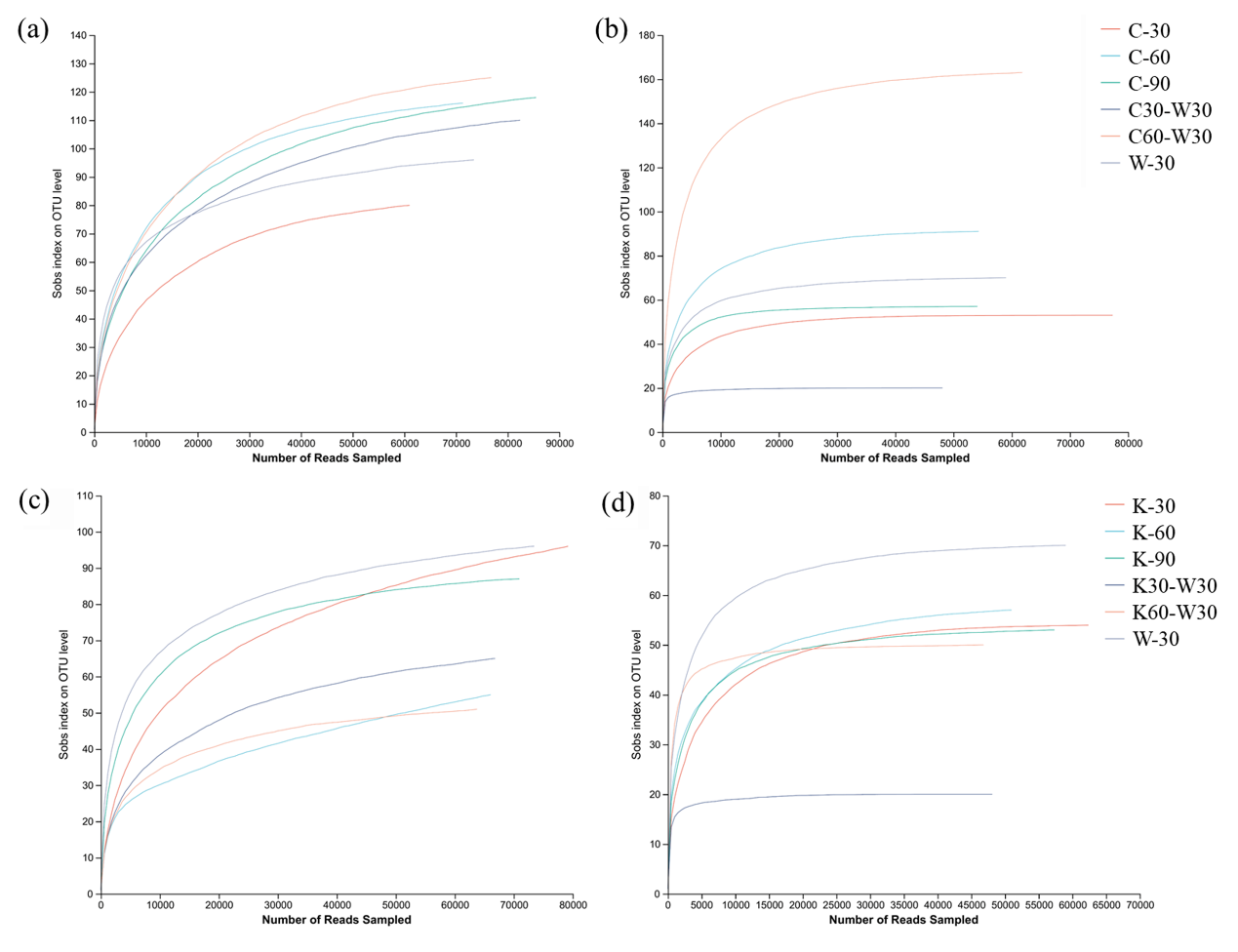
**

**Supplementary Figure S3. Rarefaction curves. (a) shows the bacterial communities of cigar in coffee group. (b) shows the fungal communities of cigar in coffee group. (c) shows the bacterial communities of cigar in cocoa group. (d) shows the fungal communities of cigar in cocoa group.**
